# High-density multi-population consensus genetic linkage map for peach

**DOI:** 10.1371/journal.pone.0207724

**Published:** 2018-11-21

**Authors:** Cassia da Silva Linge, Laima Antanaviciute, Asma Abdelghafar, Pere Arús, Daniele Bassi, Laura Rossini, Stephen Ficklin, Ksenija Gasic

**Affiliations:** 1 Clemson University, Department of Plant and Environmental Sciences, Clemson, SC, United States of America; 2 Institut de Recerca i Tecnologia Agroalimentàries (IRTA), Centre de Recerca en Agrigenòmica Consejo Superior de Investigaciones Científicas (CSIC)-IRTA–Universitat Autònoma de Barcelona (UAB)–University of Barcelona (UB), Campus UAB, Bellaterra (Cerdanyola del Vallès), Barcelona, Spain; 3 Università degli Studi di Milano, Department of Agricultural and Environmental Sciences–Production, Landscape, Agroenergy, Milan, Italy; 4 Washington State University, Department of Horticulture, Pullman, WA, United States of America; Texas Tech University, UNITED STATES

## Abstract

Highly saturated genetic linkage maps are extremely helpful to breeders and are an essential prerequisite for many biological applications such as the identification of marker-trait associations, mapping quantitative trait loci (QTL), candidate gene identification, development of molecular markers for marker-assisted selection (MAS) and comparative genetic studies. Several high-density genetic maps, constructed using the 9K SNP peach array, are available for peach. However, each of these maps is based on a single mapping population and has limited use for QTL discovery and comparative studies. A consensus genetic linkage map developed from multiple populations provides not only a higher marker density and a greater genome coverage when compared to the individual maps, but also serves as a valuable tool for estimating genetic positions of unmapped markers. In this study, a previously developed linkage map from the cross between two peach cultivars ‘Zin Dai’ and ‘Crimson Lady’ (ZC^2^) was improved by genotyping additional progenies. In addition, a peach consensus map was developed based on the combination of the improved ZC^2^ genetic linkage map with three existing high-density genetic maps of peach and a reference map of *Prunus*. A total of 1,476 SNPs representing 351 unique marker positions were mapped across eight linkage groups on the ZC^2^ genetic map. The ZC^2^ linkage map spans 483.3 cM with an average distance between markers of 1.38 cM/marker. The MergeMap and LPmerge tools were used for the construction of a consensus map based on markers shared across five genetic linkage maps. The consensus linkage map contains a total of 3,092 molecular markers, consisting of 2,975 SNPs, 116 SSRs and 1 morphological marker associated with slow ripening in peach (SR). The consensus map provides valuable information on marker order and genetic position for QTL identification in peach and other genetic studies within *Prunus* and Rosaceae.

## Introduction

A genetic linkage map represents positions and genetic distances of molecular markers on chromosomes allocated based on segregation data and recombination events of individuals in a mapping population [1,2]. Genetic maps are important tools for a vast number of genetic applications and are widely used in plant breeding programs, genetics and genomics studies. In particular, these maps are crucial for a better understanding of marker-trait associations through quantitative trait loci (QTL) mapping, discovery of genes associated with economically important fruit quality and disease resistance traits, and successful deployment of molecular markers in plant breeding programs via marker-assisted selection (MAS) [3–5]. In addition, linkage maps provide an important foundation for other biological applications including candidate gene identification, map-based gene cloning, genome evolution, comparative genomics studies and genome assembly [6–11]. High-resolution maps which cover the entire genome with co-segregating, reproducible and high-throughput markers at short intervals are most valuable because of the increased resolution that leads to more effective QTL mapping, candidate gene detection, and more precise estimates of QTL effect [5, 12,13].

Peach is a recognized model for Rosaceae genetics and genomics with a wealth of publicly available resources [14,15]. Recent advances in next-generation high-throughput sequencing and genotyping techniques, such as development of the IPSC 9K peach array [16], have permitted rapid development of high-quality genetic linkage maps [17–20].

In peach (*Prunus persica* L. Batsch), linkage maps have been used in QTL discovery of physiological traits, key fruit quality traits such as fruit size, diameter, firmness, acidity, individual sugars (fructose, glucose, sucrose and sorbitol), aroma, flesh, peel related phenolic compounds, and disease resistance traits [17,18,21–31]. These maps were typically developed for mapping particular traits in specific parental backgrounds and differ in population size and molecular markers used [4,5] resulting in limited value for comparative studies.

Multiple maps developed for the same species usually contain many common markers, which can be used as anchor points for consensus map integration [4,5,32,33]. Highly saturated genetic maps with evenly distributed markers across linkage groups, with no regions of low marker density are most suitable for the construction of a consensus map. Consensus maps developed from multiple populations provide a higher marker density and a greater genome coverage when compared to the individual maps. They also serve as valuable tools for estimating genetic positions, detecting inconsistencies among maps, comparing marker distributions and QTL locations [5]. Consensus maps could also aid estimation of genetic positions of unmapped markers (markers without genetic position) included in genotyping arrays. This is especially important in pedigree-based QTL analyses [34] that require precise genetic positions of the markers to accurately detect QTLs in pedigree-related individuals, when development of mapping populations is improbable. To assign genetic positions to unmapped markers, the common approach was to use a genome-wide mean as a conversion factor [35]. In order to overcome the problem of using the static conversion factor, Fresnedo et al. [30] developed a consensus RosBREED [36] linkage map (RC^1^) for peach predicting genetic distances by incorporating the physical and genetic positions of 68 markers from the *Prunus* bin map [37]. However, this map was developed by calculating genetic positions using polynomial equations, not by merging individual peach linkage maps.

In the Rosaceae family, a consensus map was developed for pear [38] and two integrated linkage maps have been reported in apple based on merging five and three populations [5,39]. Although a peach consensus map was previously reported [25], it was constructed using only two peach linkage maps and the GoldenGate genotyping platform which is less commonly used in the peach community compared to the IPSC 9k SNP array.

Consensus maps built using freely available MergeMap [40] and LPMerge algorithms [41] were reported in different plants, including barley [42,43], cowpea [44], rapeseed [45], spring wheat [46], loblolly pine [47], cassava [48], oak [49], oilseed rape [50] and faba bean [51], pear [38], and two integrated linkage maps in apple [5, 39].

In this study, we report the improvement of the previously developed peach linkage map ‘Zin Dai’ x ‘Crimson Lady’ (ZC^2^) by genotyping additional progenies. In addition, a consensus peach linkage map was created based on the improved ZC^2^ map and four other unrelated high-density maps using two algorithms (MergeMap and LPMerge). The consensus map provides valuable information on marker order and genetic position and will be useful in future studies of pedigree-based QTL analyses in peach.

## Materials and methods

### Plant material and DNA extraction

An F_2_ mapping population obtained from selfing an individual from the cross between ‘Zin Dai’ and ‘Crimson Lady’ (ZC^2^) was previously reported [17]. A map was elaborated based on 25 selected seedlings, genotyped with the 9k peach SNP array [16]. In this paper, we have genotyped an additional set of 65 individuals (for a total of 90 individuals) for the development of an improved genetic linkage map. DNA was isolated from young and healthy leaf tissue as described previously by Dellaporta et al. [52]. The concentration and purity of DNA was measured by a NanoDrop ND-1000 spectrophotometer. The final concentrations of all DNA samples were adjusted to 50 ng/μl for high-throughput genotyping.

### Genotypic data

DNA samples for a total of 65 ‘Zin Dai’ × ‘Crimson Lady’ seedlings and parental genotypes were submitted to the Research Technology Support Facility at Michigan State University (East Lansing, MI, USA) for genotyping by the peach Illumina 9K SNP array v1. The iScan data output files were analyzed as previously described by Frett et al. [17]. Briefly, the GenomeStudio software was used to verify the quality for all samples and SNPs observed. Markers with GenTrain score above 0.4 were inspected. The failed and monomorphic markers were excluded, whereas the polymorphic SNPs were further inspected for clustering analysis. Markers with more than three expected clusters (AA, AB and BB) and missing in at least one of the parental genotypes were excluded from further analysis. SNP markers for which the number of missing genotypes was greater than 10% were not considered for map construction.

### SNP-based linkage map construction

The improvement of the existing SNP-based genetic linkage map was based on combining polymorphic SNP marker data, observed in this study, with previously mapped marker data from the ZC^2^ mapping population [17]. A genetic linkage map was constructed using SNPs homozygous for alternate allele in two grandparents (AA in one parent and BB in other) as well as SNPs homozygous in one and heterozygous in the other grandparent. F_2_ population type codes were applied [53].

SNP markers mapped to the same location, identical markers, were grouped into single bins with the purpose of reducing map complexity for linkage analysis. A single SNP containing no missing data for a progeny was used for linkage analysis from each bin.

Linkage map construction was performed by the JoinMap 4.1 (Kyazma, NL) software applying Maximum Likelihood (ML) function [53]. The parameters used for map construction were as follows: a minimum of a logarithm of the odds (LOD) score of 3.0 was used to assign markers to linkage groups with a maximum recombination fraction of 0.4, goodness-of-fit jump threshold of 5.0 and a triplet threshold of 1.0. Markers exhibiting segregation distortion were identified applying the Chi-square (X^2^)-goodness-of-fit test (p < 0.05) and also integrated into the map. Graphical presentation of an improved SNP-based genetic linkage map of the ZC^2^ progeny consisting of eight linkage groups was generated by MapChart version 2.3 software [54]. Marker genetic distances on the linkage groups were presented in centimorgans (cM).

### Comparison of an improved ZC^2^ linkage map with the peach genome sequence v2.0

The genetic positions of each SNP marker mapped to the ZC^2^ linkage map was aligned with their physical position on the peach genome v2.0 sequence [55] by MapChart 2.3 [54], similarly to what had been previously described by Frett et al. [17].

### Consensus map construction

Genetic distances of SSR and SNP markers, as well as slow ripening locus (*Sr*), mapped across four integrated F_2_ linkage maps: PI91459(‘NJ Weeping’) × ‘Bounty’ (WB) [18], ‘O’Henry’ × ‘Clayton’ (OC) [19], ‘Venus’ × ‘Venus’ (VxV) [20] and ‘Texas’ × ‘Earlygold’ (T×E) [56] were obtained from the Genome Database for Rosaceae (GDR) [14–15]. The MergeMap software [40] and LPmerge R package [41] were used to merge four previously reported genetic linkage maps with the improved (ZC^2^) map developed in this study. To prepare input data for Mergemap and LPmerge, the SNP markers that were non-collinear in comparison with the peach genome were removed from individual maps. For LPmerge, the maximum interval parameter K varied from 1 to 4, and the composite map with the lowest root mean square error (RMSE) was selected. The consistency of all marker names across five linkage maps was verified to avoid marker duplications on the consensus map. The consensus map was constructed by merging a single linkage group (LG) of all five maps at the time, following the protocol reported by Khan et al. [5]. A weight of 1.0 was applied to all linkage groups across all maps. The RMSE in marker order between the consensus maps and the input maps, were calculated by the R package hydroGOF [57], as described in Westbrook et al. [47], and the consensus map with the lowest average RMSE was used for further analysis. The physical positions of all markers mapped to the consensus peach linkage map were compared to the peach genome sequence v2.0 [55] and visualized in Mapchart 2.0 [54].

### Estimating the genetic position (cM) for unmapped SNP markers in the 9K SNP array

A Perl Script was developed to estimate the genetic positions for the unmapped SNP markers in the 9K SNP array using the peach consensus map as a reference. The term “unmapped” designates the markers from the genotyping array that were not mapped in one of the individual maps used for building the consensus map. The genetic position for each unmapped marker was estimated using the two closest mapped SNPs in the peach consensus map reported in this study. The equations are as follows:

delta_bp=snp2_bp−snp1_bp

delta_cM=snp2_cM−snp1_cM

cM_estimate=snp1_cM+delta_cM*(snp_bp−snp1_bp)/delta_bp)

where: delta_bp is distance in bp between mapped SNPs in the consensus map; delta_cM is distance in cM between mapped SNPs in the consensus map; snp_bp is the physical position of the peach SNP being estimated (in bp); snp1_bp and snp2_bp are the immediate left and right physical positions (bp) of SNPs that map to the genetic map and snp1_cM and snp2_cM are their corresponding genetic positions (in cM).

In cases where a SNP was beyond the last mapped SNP, the same delta_cM from the last two SNPs on the linkage group and snp2_bp became the position at the scaffold end.

## Results

### The improved linkage map for ZC^2^ population

**Fig 1:**
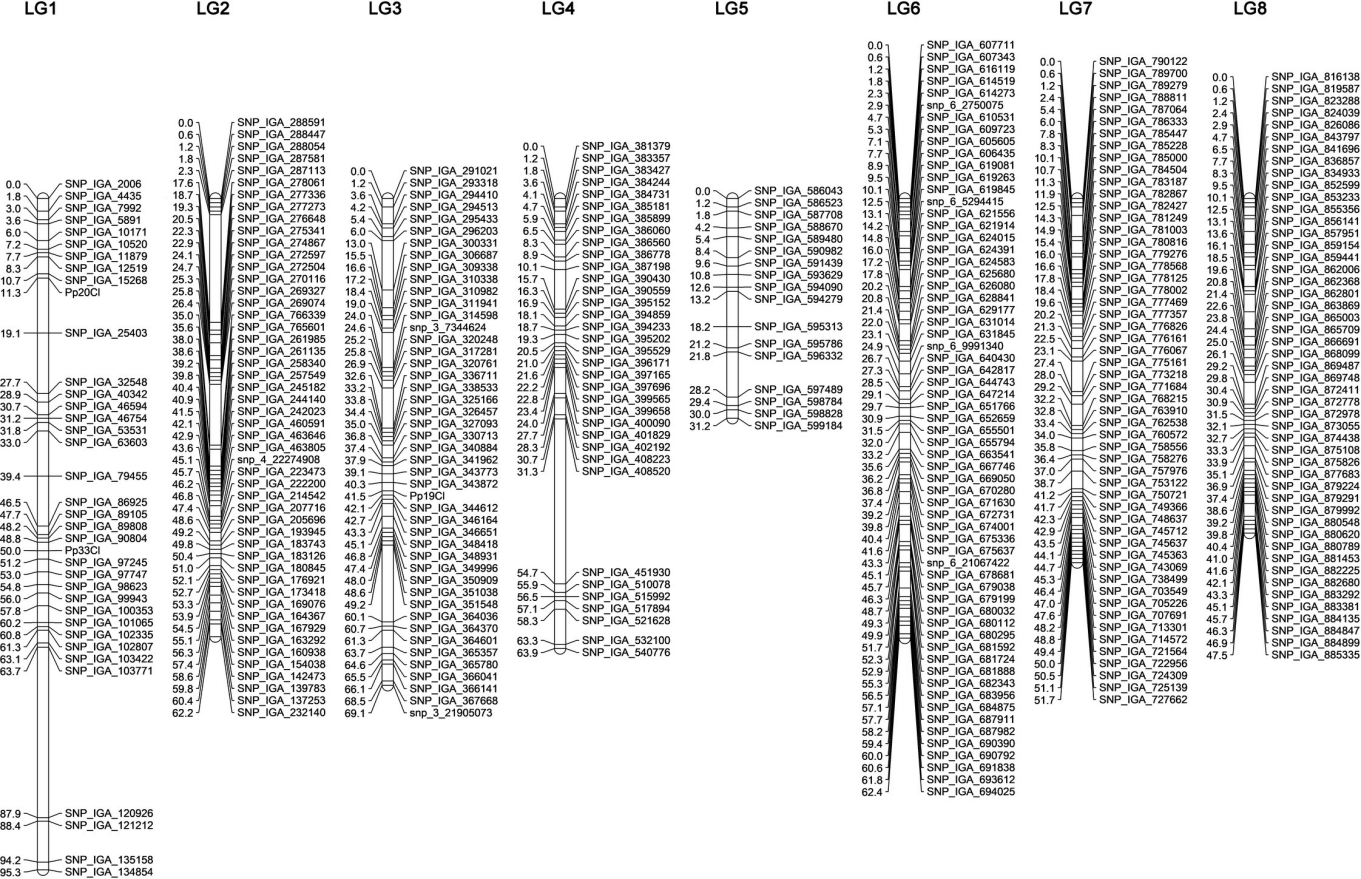
The improved SNP-based genetic linkage map of ‘Zin Dai’ × ‘Crimson Lady’ (ZC^2^) progeny. Marker names are listed at the right side of each LG and the genetic position (in cM) is listed at the left of each marker.

The construction of the improved SNP-based linkage map was based on heterozygous SNPs observed in this study combined with SNP marker data previously reported by Frett et al. [17]. A total of 1,478 SNPs were informative in the ZC^2^ progeny. Out of those, 2 SNPs were unlinked (0.1%) and 1,476 were used for map construction. Maximum Likelihood mapping successfully mapped 1,476 SNP markers with 351 unique positions (S1 Table; Fig 1).

The revised linkage map of the ZC^2^ progeny spanned a total genetic distance of 483.3 cM, with linkage group 1 (LG1) being the longest (95.3 cM) and LG5 the shortest (31.2 cM). The highest number of SNPs mapped to a single linkage group was 263 on LG7 and the lowest was 40 on LG5. The number of unique map positions mapped on a single linkage group ranged from 63 on LG6 to 17 on LG5. The largest gap was observed in LG1 (24.2 cM) between SNP_IGA_103771 and SNP_IGA_120926 (Table 1). SNP marker density per linkage group ranged from 0.96 to 2.58 cM with the average of 1.38 cM.

**Table 1:** The improved SNP-based genetic linkage map of ‘Zin Dai’ × ‘Crimson Lady’ (ZC^2^) progeny.

LG	Length (cM)	Mapped markers	Uniquely mapped	SNPs mapped to the same position	Largest gap (cM)
LG1	95.3	131	37	94	24.2
LG2	62.2	259	50	209	15.3
LG3	69.1	161	46	115	10.9
LG4	63.9	211	35	176	23.4
LG5	31.2	40	17	23	2.6
LG6	62.4	234	63	171	2.4
LG7	51.7	263	54	209	4.3
LG8	47.5	177	49	128	6.4
ZC^2^ map	483.3	1,476	351	1,125	

### Comparison of the ZC^2^ linkage map with the peach physical map v 2.0

**Fig 2:**
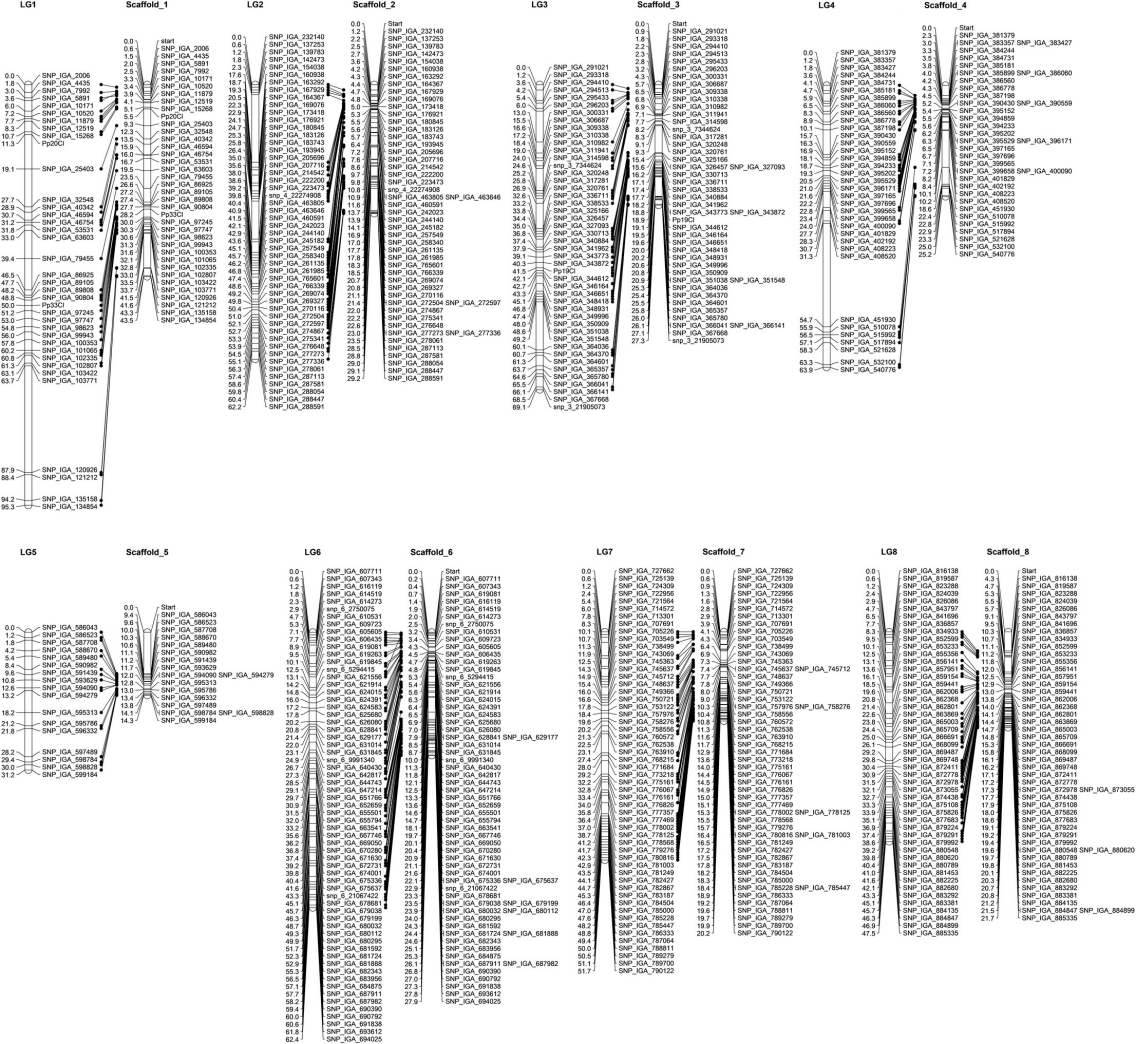
Alignment of the ZC^2^ linkage map and the peach genome sequence v2.0. Peach genome scaffolds and ZC^2^ linkage groups are shown on the left and right of each pair, respectively.

The ZC^2^ map covers approximately 82.7% of the peach genome v2.0 (Table 2). LG3 had the largest coverage (96%), while the lowest coverage (26%) was observed on LG5. The improved ZC^2^ genetic map had 97.8% of all SNP markers in agreement with their positions on the scaffolds of the peach genome v 2.0 with differences in the marker order identified in LGs 1, 2, 3 and 6 (Table 2, Fig 2). LG3 had the highest number of non-collinear SNP markers (28). The recombination rate of different chromosomes was estimated as the quotient between the genetic distance (cM) covered by the corresponding LG and the size in Mb of the chromosome fragment covered with markers. This value ranged from 2.20 cM/Mb on LG6 to 6.53 cM/Mb on LG5, almost a three-fold difference in the recombination rate of the corresponding genomic regions (Table 2).

**Table 2:** Comparison of the ZC^2^ linkage map with the peach physical map v 2.0.

LGs	ZC^2^ linkage map					Marker density		Ratio (cM/Mb)^2^
	# of SNPs (non-collinear)	# of bins^1^	Genetic distance (cM)	Physical length (Mb)	Physical coverage (%)	cM	Mb	
1	131 (1)	37	95.3	43.04	90.2	2.57	1.16	2.21
2	259 (3)	50	62.2	28.07	92.8	1.24	0.56	2.21
3	161 (28)	46	69.1	26.39	96.8	1.50	0.57	2.63
4	211 (-)	35	63.9	23.13	91.2	1.82	0.66	2.75
5	40 (-)	17	31.2	4.83	26.5	1.83	0.28	6.53
6	234 (1)	63	62.4	28.21	92.9	0.99	0.45	2.20
7	263 (-)	54	51.7	20.77	93.2	0.95	0.38	2.50
8	177 (-)	49	47.5	17.41	77.8	0.96	0.36	2.66

^1^ Groups of markers with the same genetic position

^2^ Ratio between genetic distance and physical length that estimates the recombination rate per chromosome

### Comparison of the two versions of the ZC^2^ map

The reconstruction of the ZC^2^ linkage map resulted in a higher number of mapped markers, from 1,335 mapped on existing map [17] up to 1,476 SNPs mapped on the improved ZC^2^ map. The number of unique SNP positions mapped increased from 190 in the previous map to 351 in the improved map. In addition, the SNP marker density in the improved map (1.38 cM/marker) was higher than that reported in the previous one (2.3 cM/marker). The improved genetic linkage map consisted of eight linkage groups, corresponding to the number of scaffolds in the peach genome, while the previous map consisted of 14 linkage groups.

### Consensus genetic map of peach

Four previously published bi-parental linkage maps, WB [18], OC [19], VxV [20], TxE [56] and an improved ZC^2^ map developed in this study, were used to construct the consensus peach map. The number of markers mapped on these maps ranged from 1,948 in TxE to 877 in WB.

SNPs that mapped to positions that are non-collinear with their physical position on the peach genome were removed from individual maps and 3,092 markers, including 2,975 SNPs, 116 SSRs and one morphological marker (SR) associated with slow ripening in peach [31] distributed in eight linkage groups, were used to build the consensus map by two different algorithms, i.e. MergeMap and LPmerge.

A total of 1,416 SNPs were common to at least two linkage maps with 2,547 anchor points (Table 3). There were 457 anchor points between VxV and TxE maps, while only 98 anchor points were observed between WB and ZC^2^ maps. LG4 had the highest number of anchors points (648), while the lowest number was detected in LG5 (100). The highest number of common markers among the LGs was observed in LG4 (325) and the lowest was observed on the LG5 with only 70 common markers.

**Table 3:** Comparison between five peach genetic maps for common markers and anchor points across different linkage groups used to construct a consensus genetic map.

Linkage Maps	LG1	LG2	LG3	LG4	LG5	LG6	LG7	LG8	Anchors/Map
WB vs. OC	51	11	35	33	6	12	33	29	210
WB vs. VxV	82	10	45	54	1	19	22	26	259
WB vs. TxE	62	24	44	38	36	37	29	33	303
WB vs. ZC^2^	14	2	9	19	13	12	14	15	98
OC vs. VxV	89	19	76	138	1	10	38	26	397
OC vs. TxE	38	66	45	110	14	20	53	38	384
OC vs. ZC^2^	10	11	17	22	10	1	42	20	133
VxV vs. TxE	75	39	65	144	1	45	32	56	457
VxV vs. ZC^2^	6	8	16	62	1	9	13	14	129
TxE vs. ZC^2^	13	15	17	28	17	25	30	32	177
Anchors/LG	440	205	369	648	100	190	306	289	2547
Number of markers	217	149	178	325	70	133	177	167	

The anchor points between pair of genetic maps and corresponding linkage groups, as well as the total number of markers in common on each linkage group are shown. WB, ‘NJ Weeping’ x ‘Bounty’ [18]; OC, ‘O’Henry’ x ‘Clayton’ [19]; VxV, ‘Venus’ × ‘Venus’ [20]; TxE, ‘Texas’ x ‘Earlygold’ [56]; ZC^2^, ‘Zin Dai’ x ‘Crimson Lady’ improved map.

### Consensus genetic maps built by MergeMap and LPmerge algorithms

Consensus maps were successfully developed by MergeMap and LPMerge algorithms. However, mismatch in marker order between the two versions of the consensus map was observed (S2 and S3 Tables). The MergeMap consensus map had a genetic distance of 830.62 cM with the length of individual LGs ranging from 86.96 to 143.95 cM, observed in LG5 and LG1, respectively (S2 Table). Average distance between the markers was 0.92 cM and the largest gap size was 8.8 cM on LG2. There were 906 uniquely mapped positions ranging from 156 on LG1 to 76 on LG5 (Table 4; S1 Fig).

**Table 4:** Comparison between peach consensus maps built using MergeMap and LPmerge.

LG	No. of Markers	# of bins^1^	LG length (cM)	SNP density
1	460	156 |121	143.95 | 96.05	0.92 | 0.79
2	406	84 | 64	97.37 | 63.48	1.16 | 0.99
3	335	118 | 59	96.64 |46.6	0.82 |0.79
4	670	132 | 86	104.22 | 64.13	0.79 | 0.75
5	182	76 | 61	86.96 | 62.44	1.14 | 1.02
6	363	119 | 113	96.57 |78.6	0.81 | 0.70
7	317	109 | 98	102.61 | 68.92	0.94 | 0.70
8	359	112 | 91	102.30 | 57.7	0.91 | 0.63
Total	3,092	906 | 693	830.62 | 537.92	0.92 | 0.78

Number of bins, linkage group length and marker density per linkage group generated by MergeMap and LPmerge are shown on left and right, respectively.

^1^ Groups of markers with the same genetic position.

**Fig 3:**
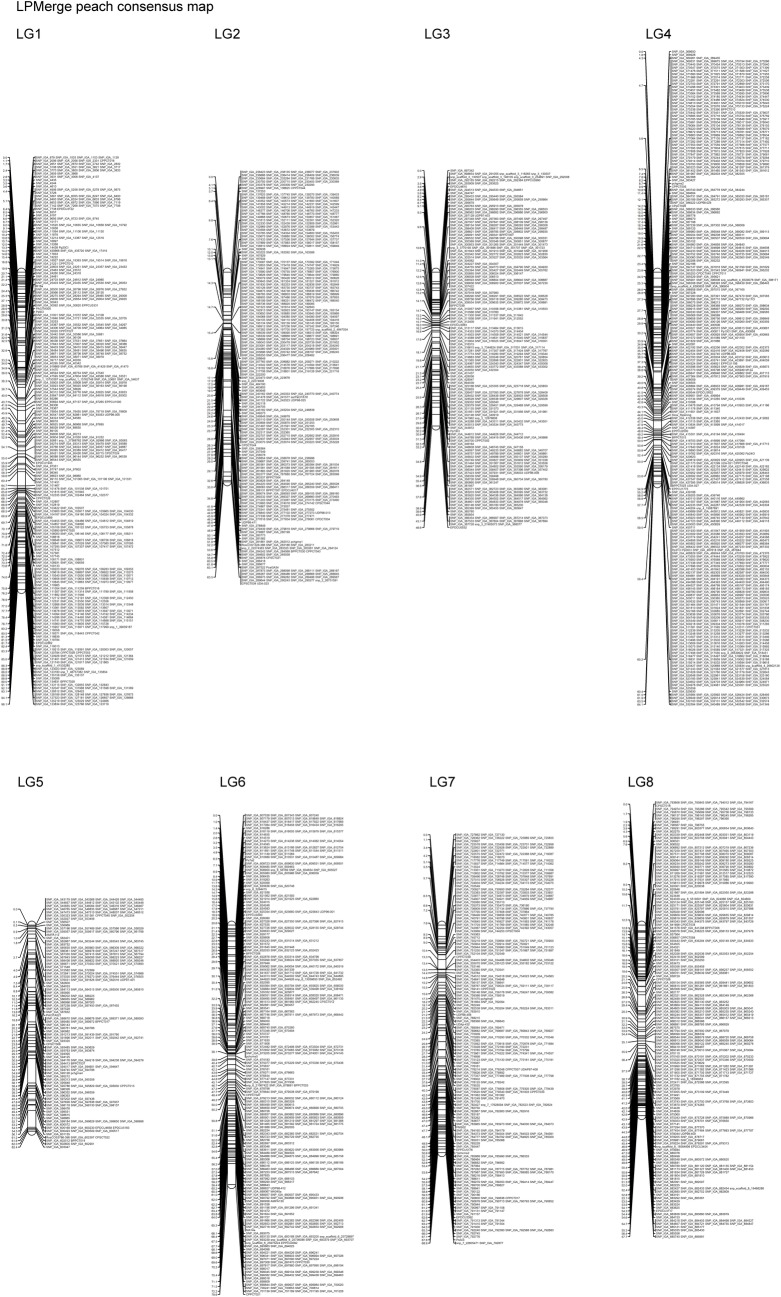
Peach consensus linkage map built using four previously published bi-parental linkage maps, PI91459(‘NJ Weeping’) × ‘Bounty’ (WB) [18], ‘O’Henry’ × ‘Clayton’ (OC) [19], ‘Venus’ × ‘Venus’ (VxV) [20] and ‘Texas’ × ‘Earlygold’ (T×E) [56], and an improved ‘Zin Dai’ × ‘Crimson Lady’^2^ map developed in this study with LPMerge algorithm. Marker names are listed at the right side of each LG and the genetic position (in cM) is listed at the left of each marker.

The consensus map built with the LPMerge algorithm spanned 537.92cM, with the length of individual LGs ranging from 46.6 to 96.05cM for LG3 and LG1, respectively (S3 Table). Average distance between the markers was 0.78 cM and the largest gap of 7.31 cM was observed on LG5 (Table 4; Fig 3). The number of uniquely mapped positions were 693, with the lowest in LG3 (59), and the largest in LG1 (121). The LPMerge peach consensus map had the lowest average RMSE and was further referred to as the peach consensus map (S4 Table).

### Comparison of the peach consensus map with the peach physical map v2.0

The physical length of the peach consensus map was estimated to cover approximately 98% of the pseudomolecules of peach genome v2.0 with most of the scaffolds having a coverage above 95%, except for scaffold 5 (91.0%). The recombination rate of different chromosomes ranged from 1.63 cM/Mb on LG4 to 3.77 cM/Mb on LG5. The consensus map was collinear with the peach genome revealing complete agreement in the SNP marker order (S2 Fig; Table 5).

**Table 5:** Comparison of the peach consensus map with the peach genome sequence v2.0.

LG	No. of Markers	Genetic distance	Physical length	Physical coverage	Marker density		Ratio
		(cM)	(Mb)	(%)	cM	Mb	(cM/Mb)^1^
1	460	96.05	47.44	99	0.79	0.39	2.03
2	406	63.48	30.13	99	0.99	0.47	2.10
3	335	46.60	27.25	99	0.79	0.46	1.71
4	670	64.13	25.15	99	0.75	0.29	2.59
5	182	62.44	16.52	91	1.02	0.27	3.77
6	363	78.60	30.10	99	0.70	0.27	2.59
7	317	68.92	22.19	98	0.70	0.22	3.18
8	359	57.70	21.91	97	0.63	0.24	2.63

^1^Ratio genetic distance/physical length that estimates the recombination rate per chromosome.

The peach consensus map was used as a reference with a Perl script (developed in-house) to calculate genetic positions of markers from the peach 9K array, and the genetic positions of 6,019 unmapped SNP markers were provided in S5 Table.

## Discussion

### The improved linkage map for ZC^2^ population

Genotyping of additional 65 F_2_ individuals from the cross ‘Zin Dai’ and ‘Crimson Lady’ improved the existing ZC^2^ map [17] and resulted in a map with a better resolution and more uniquely mapped positions. In comparison to the previous ZC^2^ map, the number of linkage groups decreased from 14 to eight and the number of mapped markers increased from 1,335 to 1.476. The number of unique positions increased by approximately 84% (Table 1) as well as marker density (from 2.4 to 1.38cM/marker). The first version of the map covered 61.6% of the pseudomolecules of the peach genome, while the improved map covered 82.7%. Genetic length (483.3 cM) and SNP density (1.38 cM/SNP) of the improved ZC^2^ map were similar to previously reported SNP maps in peach [25,28,19]. The new ZC^2^ map had a higher marker density than the other maps based on the 9K SNP array [28,19]. The observed gaps on LGs 1 and 6 (24.2 and 23.4 cM, respectively) agreed with those reported by Yang et al. [19] and Frett et al. [17] who used the same genotyping strategy.

Marker order comparison between the ZC^2^ genetic map and the physical map, based on peach genome v2.0, revealed discrepancies in marker positions across LGs 1, 2, 3 and 6. Non-collinearity in other peach maps has been reported when both the peach genome v1 [24–25,17,18] and v2.0 [28] were used for comparison. Non-collinearity in marker order could be due to specific characteristics of the population, such as size, presence of chromosome rearrangements, and/or linkage mapping and genotyping errors. It could also indicate misassemblies in the peach genome sequence v2.0 [55]. The improved ZC^2^ map provides an excellent resource for mapping QTLs associated with fruit quality and phytochemical compounds, since the ZC^2^ progeny segregate for many traits including flowering and ripening time, blush, fruit size, flesh adhesion and texture, and phytochemical content [58]. Thus, the improved ZC^2^ map provides a valuable tool for future work to better understand genetic mechanisms that control these traits in peach.

### Consensus genetic map of peach

The peach research community has been using a *Prunus* genetic map based on an interspecific cross between almond ‘Texas’ and peach ‘Earlygold’ (TxE) [3,56,59] as a reference for establishing linkage group orientation and comparative QTL studies. Prior to the availability of the peach genome sequence, the TxE map was a valuable tool as a source of mapped and transferable markers (mainly SSRs and RFLPs) for the construction of low density maps and the comparison between intraspecific peach and other *Prunus* species maps [59]. The release of the peach genome sequence [55,60] triggered the development of the 9K peach SNP array [16] and promoted genetic studies in peach using a common genotyping strategy [17,18,19,20,56]. This established the foundation for the development of the peach consensus map reported in this study.

The five highly saturated maps used for building the consensus peach map were based on SSR and SNP markers [20,56] or exclusively SNP markers [17,18]. The high number of common markers (1,416) and anchor points (2,547) facilitated the integration of the individual linkage maps into the consensus map and provided reliable information about SNP marker order and genetic distance in the consensus map. The number of anchor points observed in the peach consensus map was higher than that observed in the consensus maps developed for apple [5,39] and pear [38].

The MergeMap algorithm resulted in consensus map with a higher genetic length (830.62cM) and a lower marker density (0.92cM/marker) compared to the LPMerge algorithm (537.92 cM and 0.78cM/marker, respectively). A possible explanation for the observed differences between the two algorithms is that the MergeMap assigned unique positions to most of the markers, while the LPMerge binned markers into the same map positions. Thus, the non-binning attribute of the MergeMap provided higher genetic length of the consensus map [47]. The overestimated genetic length in the consensus map constructed by the MergeMap was previously reported in pear [38], barley [43] *Pinus taeda*, and *Pinus elliottii* [47]. On the other hand, the genetic length of the LPMerge peach consensus map was within the range of the five individual maps used in this study (336.0–536.6 cM). In addition, each algorithm ordered markers differently in the consensus map resulting in non-collinearity in the MergeMap peach consensus map with peach genome v2.0. A possible explanation is that MergeMap simplified consensus graphs were not ordinally equivalent to the original linkage maps used for building the consensus map [61]. The LPMerge map had the lowest RMSE compared to the input maps and was chosen as the consensus map.

The peach consensus map described here exhibited approximately 98% coverage and full SNP collinearity with the pseudomolecules/scaffolds of the peach genome v2.0 [55], which is similar to coverage obtained with consensus maps developed for apple [5] and pear [38]. The high level of genome coverage confirms the correct positioning of the markers in the consensus map that emerges as reliable tool for future genetic studies such as QTL mapping and candidate gene analyses [5].

This is, to our knowledge, the most comprehensive peach consensus map constructed thus far. Although two consensus peach maps have been previously reported, their application is limited due to either small number of genotypes providing recombination events and less common genotyping platform in the peach community [25], or being developed not by merging individual peach linkage maps but by calculating genetic positions [30]. The consensus map reported in this study is an alternative source of information for calculating genetic positions of unmapped markers in the 9K peach SNP array and QTL mapping via pedigree [34].

## Conclusions

In this study, we genotyped 65 additional F_2_ individuals using the 9K SNP array and significantly increased the resolution of the previously published ZC^2^ map. Using the improved ZC^2^ map with four other high-density linkage maps (all genotyped with the 9K SNP array), we developed a high-resolution consensus map for peach using LPMerge algorithm. The peach consensus linkage map contains a total of 3,092 molecular markers (2,975 SNPs, 116 SSRs and 1 morphological marker associated with slow ripening in peach), 2,547 anchor points and covers approximately 98% of the physical length of the peach genome v2.0. This consensus genetic linkage map represents the most comprehensive peach map available to date and could serve as a new reference map for peach. The consensus map provides valuable information on marker order and genetic position for QTL identification and molecular marker development in peach and other genetic studies within the *Prunus* and Rosaceae.

## Supporting information

S1 Fig MergeMap peach consensus map with 3,092 markers. Marker names are listed at the right side of each LG and the genetic position (in cM) are listed at the left of each marker.
(TIF)

S2 Fig Alignment of the peach consensus map and the peach genome sequence v2.0. Peach genome scaffolds and linkage groups are shown on the left and right of each pair, respectively.
(TIF)

S1 Table The improved SNP-based genetic linkage map of ‘Zin Dai’ × ‘Crimson Lady’ (ZC^2^) progeny. (XLSX)

S2 Table Peach consensus map with 3,092 molecular markers and constructed using MergeMap algorithm. (XLSX)

S3 Table Peach consensus map with 3,092 molecular markers and constructed using LPMerge algorithm. (XLSX)

S4 Table Root mean squared error (RMSE) in marker order between the MergeMap and LPmerge peach consensus maps and the five input maps. (XLSX)

S5 Table Estimated genetic position of the SNPs markers from 9K SNP array using peach consensus map as a reference. (XLSX)
