# Supplementary figures and images for: High-density multi-population consensus genetic linkage map for peach
Source: PLoS One. 2018 Nov 21;13(11):e0207724. doi: 10.1371/journal.pone.0207724 (PMC6248993; doi:10.1371/journal.pone.0207724)

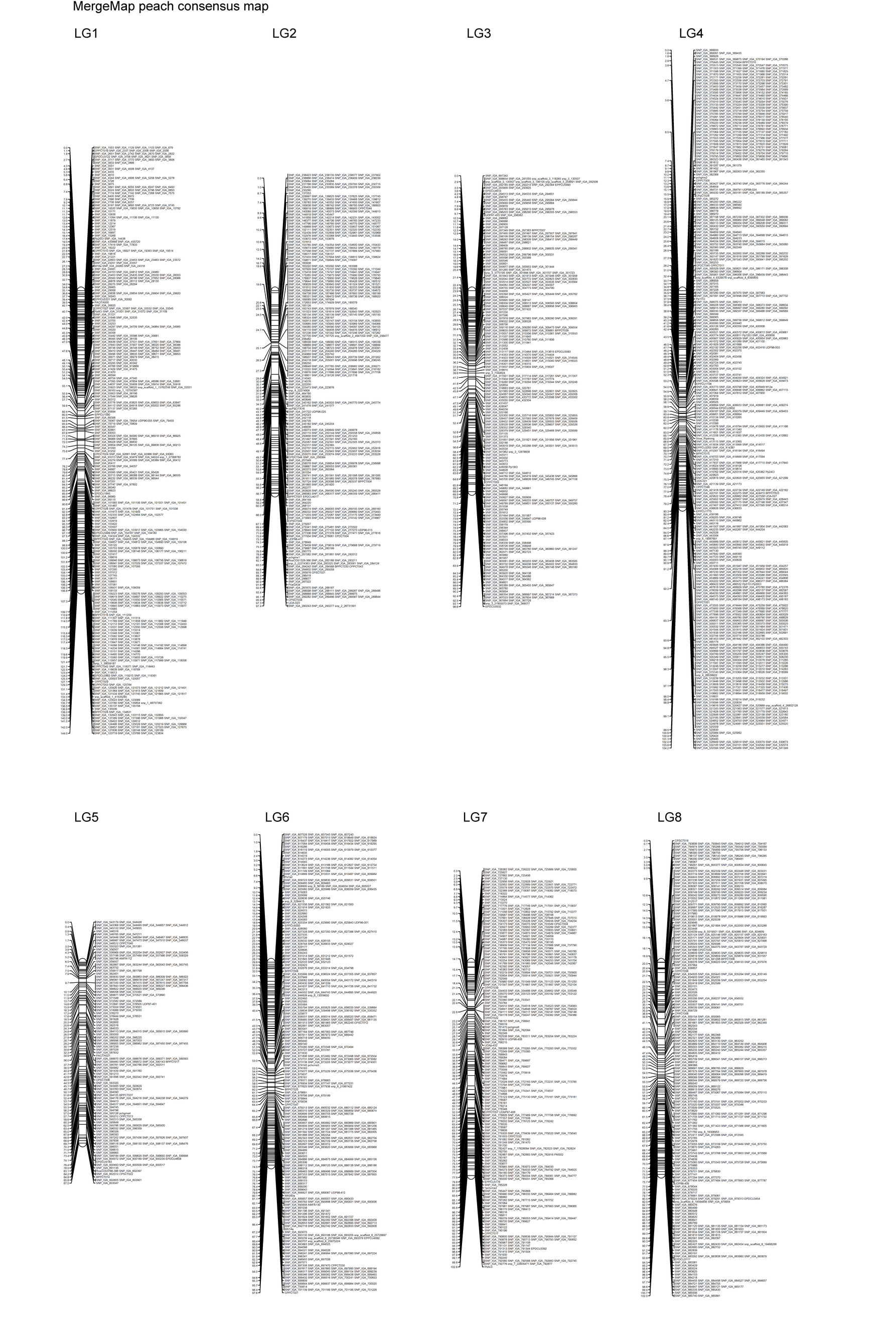

Supplement: S1 Fig — Marker names are listed at the right side of each LG and the genetic position (in cM) are listed at the left of each marker. (TIF) [file pone.0207724.s001.tif]

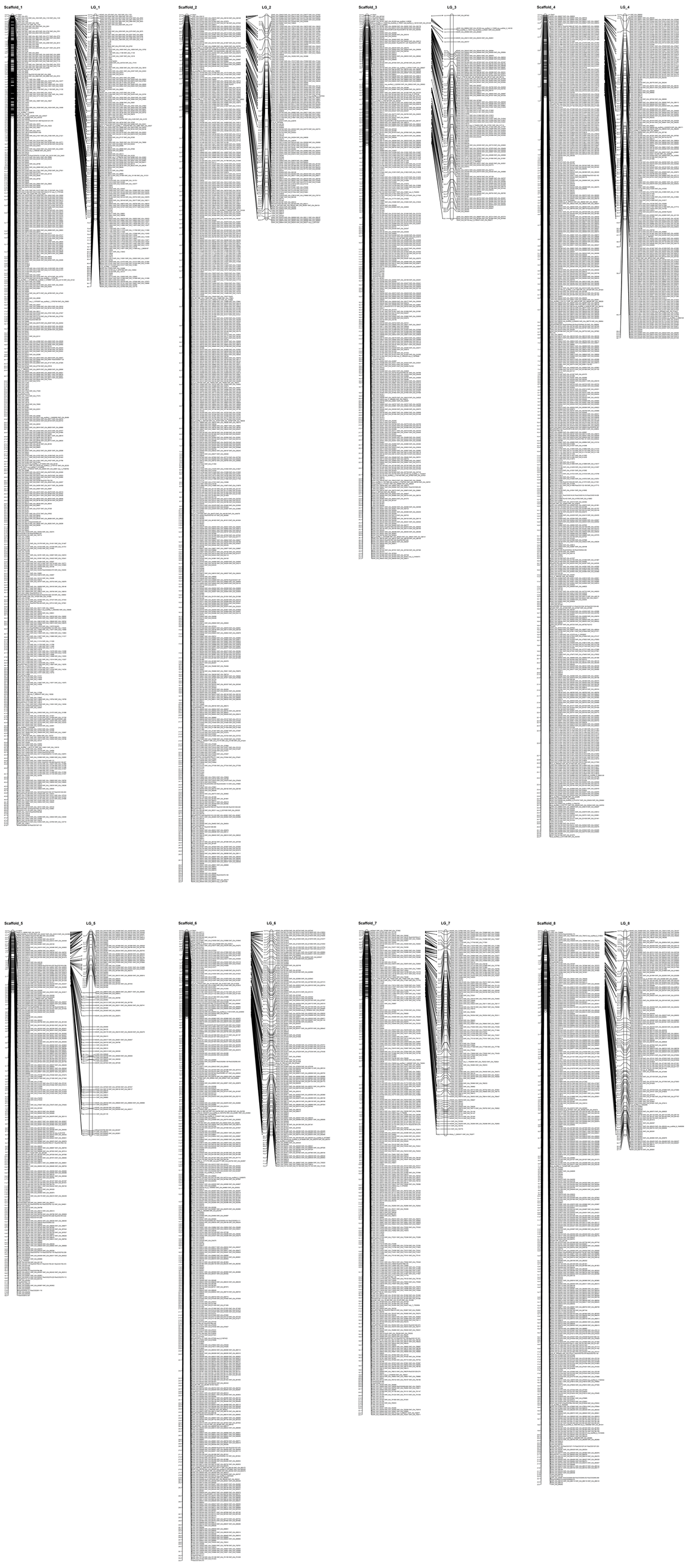

Supplement: S2 Fig — Peach genome scaffolds and linkage groups are shown on the left and right of each pair, respectively. (TIF) [file pone.0207724.s002.tif]
